# Clinical features as predictors of histologically confirmed inflammation in patients with lumbar disc herniation with associated radiculopathy

**DOI:** 10.1186/s12891-020-03590-x

**Published:** 2020-08-21

**Authors:** Jon J. Ford, Omar Kaddour, Michael Gonzales, Patrick Page, Andrew J. Hahne

**Affiliations:** 1 College of Science, Health & Engineering, La Trobe University, Bundoora, Victoria 3085 Australia; 2 Back in Form Physiotherapy, Ascot Vale, Victoria Australia; 3 The Royal Melbourne Hospital, Parkville, Victoria Australia; 4 Box Hill Radiology, Epworth Eastern Hospital, Box Hill, Victoria Australia

**Keywords:** Inflammation, Back pain, Clinical predictors, Lumbar disc herniation, Radiculopathy

## Abstract

**Background:**

An understanding of the clinical features of inflammation in low back pain with or without leg symptoms may allow targeted evaluations of anti-inflammatory treatment in randomised-controlled-trials and clinical practice.

**Purpose:**

This study evaluated the diagnostic accuracy of clinical features to predict the presence/absence of histologically confirmed inflammation in herniated disc specimens removed at surgery in patients with lumbar disc herniation and associated radiculopathy (DHR).

**Study design:**

Cohort Study.

**Methods:**

Disc material from patients with DHR undergoing lumbar discectomy was sampled and underwent histological/immunohistochemistry analyses. Control discs were sampled from patients undergoing surgical correction for scoliosis. Baseline assessment comprising sociodemographic factors, subjective examination, physical examination and psychosocial screening was conducted and a range of potential clinical predictors of inflammation developed based on the existing literature. Multi-variate analysis was undertaken to determine diagnostic accuracy.

**Results:**

Forty patients with DHR and three control patients were recruited. None of the control discs had evidence of inflammation compared to 28% of patients with DHR. Predictors of the presence of histologically confirmed inflammation included back pain < 5/10, symptoms worse the next day after injury, lumbar flexion range between 0 and 30° and a positive clinical inflammation score (at least 3 of: constant symptoms, morning pain/stiffness greater than 60-min, short walking not easing symptoms and significant night symptoms). The model achieved a sensitivity of 90.9%, a specificity of 92.9%, and a predictive accuracy of 92.3%.

**Conclusion:**

In a sample of patients with lumbar DHR a combination of clinical features predicted the presence or absence of histologically confirmed inflammation.

**Clinical relevance:**

These clinical features may enable targeted anti-inflammatory treatment in future RCTs and in clinical practice.

## What is known about the subject

- Evidence suggests that inflammatory processes are a potential treatment target for people with LBP.
- There is limited evidence for the effectiveness of anti-inflammatory treatment in LBP.
- A method of detecting patients with LBP and associated inflammation is likely required so their treatment can be tailored appropriately.

## What this study adds to existing knowledge

- A composite clinical inflammation score predicted histological inflammation in discs from patients undergoing lumbar discectomy.
- Control disc specimens had no histological evidence of inflammation.
- These clinical features may enable targeted anti-inflammatory treatment in future RCTs and in clinical practice.

## Introduction

Biologically, the degenerated lumbar intervertebral disc is a potential contributor to low back pain with or without leg symptoms (LBP) [4]. The mechanisms resulting in disc related pain are not completely understood, however the role of inflammation in disc degeneration and pain generation is supported by significant evidence [1, 33]. Studies investigating people with LBP have shown pathoanatomical changes in lumbar discs which are not observed in non-painful degenerative discs [32]. Studies have also identified the presence of inflammatory markers in people with painful degenerative discs and disc herniation with associated radiculopathy (DHR) [43] seen histologically in disc tissue [15, 17, 38, 45], using other inflammatory markers in disc tissue [5, 22] and measured by serum biomarkers [25]. Furthermore, the high serum tumour necrosis factor in acute LBP was recently demonstrated to be a predictor for poor recovery of pain and activity limitation at 6-months [26].

These findings indicate that inflammatory processes are a plausible treatment target for people with LBP treated in clinical practice or clinical trials. However, there is limited evidence for the effectiveness of anti-inflammatory treatment in LBP including epidural corticosteroid injections [35], non-steroidal anti-inflammatory drugs (NSAIDs) [8, 36, 37] and oral corticosteroids [14, 27, 35] with short term and small effects predominating. RCTs investigating the effectiveness of anti-inflammatory treatments have not selected patients based on the presence of an inflammatory contribution to LBP. In these trials, if a substantial proportion of the sample do not have an inflammatory component to their LBP, then any effect of anti-inflammatory treatment on the sample overall has the potential to be diluted. To rectify this problem, a method of detecting patients with LBP and associated inflammation would be required so treatment can be tailored accordingly.

A number of credible studies have validated clinical features of inflammatory back pain (IBP) particularly for spondyloarthropathy [2, 24, 39, 42] which include age < 40 years, insidious onset, morning stiffness, improvement with exercise, no improvement with rest and pain at night with improvement upon getting up from bed [49]. However, IBP involves different mechanisms and is therefore likely to be a different condition in comparison to disc related LBP with an inflammatory contribution. There is some data on clinical features of disc related LBP and associated inflammation, however no studies have investigated the diagnostic accuracy of clinical features in predicting the presence of confirmed inflammation [38, 45].

An understanding of the clinical features of inflammation in LBP has the potential to allow more targeted treatment clinically and more precise evaluations of anti-inflammatory effectiveness in RCTs. Therefore, this prospective study aimed to evaluate the diagnostic accuracy of clinical features to predict the presence/absence of histologically confirmed inflammation in herniated disc specimens removed at surgery in patients with DHR. As part of this aim the presence of histologically confirmed inflammation in herniated disc tissue was compared to control disc specimens from patients having surgical correction for scoliosis. Our hypothesis is that a multi-variate model based on clinical features would demonstrate high levels of sensitivity and specificity in predicting histologically confirmed inflammation.

## Materials and methods

### Study population

Ethics approval for the study was obtained from The University of Melbourne and relevant hospital ethics committees. Eleven orthopedic surgeons and neurosurgeons from Epworth Healthcare Richmond, Melbourne Health and Freemasons Hospitals in Victoria, Australia participated in the study. Patients were consecutively identified from those booked for surgical correction of scoliosis or lumbar discectomy and verbal/written informed consent was obtained.

Patients were eligible if they were literate in spoken and written English, had undergone magnetic resonance imaging (MRI) of the lumbar spine in the past 6-months, were booked in for lumbar discectomy or surgical correction of scoliosis and were willing to donate disc tissue specimens removed during surgery. Patients were excluded if they had computerized tomography scan or MRI confirmed central or lateral canal stenosis, spondylolisthesis/spondylosis or symptoms due to non-mechanical pathology (e.g. tumour, infection, inflammatory arthritis) or previous surgery to the lumbar spine. Control patients were excluded if they had received treatment for LBP in the previous 12-months due to the potential risk of having inflammatory markers in their disc tissue.

### Tissue collecting procedure

The study surgeons had an average of 10-years’ experience in spinal surgery in major private and public teaching hospitals in Australia /overseas and regularly performed discectomy and/or scoliosis correction as part of their clinical practice.

Discectomy was performed with patients in prone. A 19-gauge needle was placed into the spinous process of the disc to be operated on and the level checked with the image intensifier. An incision was made over the spinous process approximately 2-3 cm in length and a standard unilateral retractor used. A variable amount of lamina and facet joint was removed together with the ligamentum flavum to gain access. The nerve root was retracted and the disc annulus incised with a number 11 blade and the herniated material removed with a pituitary rongeur. As much herniated disc tissue as possible was collected from each patient with a particular focus around the nerve root where inflammation is likely to predominate. Sequestered disc material was not sampled. Specimens with formalin were placed in a coded container to de-identify the patient, then transported to the Department of Anatomical Pathology at The Royal Melbourne Hospital (Melbourne, Australia) for histological and immune-histochemical analyses.

### Histological analysis

Specimens were fixed in formalin for a minimum of 24-h and then routinely processed for liquid paraffin embedding at a temperature of 58 °C. Preparation for paraffin embedding was conducted by first dehydrating the tissue progressively in graded alcohols of 60, 65, 95, 95 and 100% alcohol. Three serial sections of 3 μm thickness was subsequently cut from paraffin blocks.

The sections of herniated and control disc tissue were stained with haematoxylin and eosin and examined under the microscope in × 200 magnification medium power fields. Only sections displaying evidence of inflammation as depicted in the histological stain underwent immunohistochemistry analysis [7]. The first five specimens underwent both histological and immunohistochemistry analysis and there were no cases where the two test results were discordant for presence of inflammatory markers.

### Immunohistochemistry analysis

Specimens with evidence of inflammation from the histological stain were analysed using standard immunohistochemistry protocols for the presence of inflammatory cells. Macrophages, B lymphocytes and T lymphocytes were identified as CD68, CD20 and CD3 positive cells respectively using DAKO^1^ inflammatory cell antibodies. Specimens were heated at 100 °C for 20-min in a citrate buffer (pH 6) as part of the heat induced antigen retrieval process on board a Vision Bio systems Bond-Max Immunostainer.^2^ The primary antibodies were then applied at the appropriate dilutions for 25-min at room temperature. The slide was rinsed again in phosphate buffered saline (PBS) for 5-min. The secondary antibody, consisting of 200 μl of Envision^3^ and 200 μl of Envision^4^ was added using the DAKO Autostainer.^5^

The slide was incubated at room temperature for 30-min and peroxidase added using a Labelled Polymer Immunoperoxidase System (DAKO catalogue number DS9713) according to the manufacturer’s instructions. The slide was again rinsed in PBS before peroxidase activity could be demonstrated by applying 3, 3-Diaminobenzidine (DAKO catalogue number Bond-DAB) for 10-min. The slide was washed using tap water and counterstained in Lillee-Mayer Haemotoxylin^6^ (blue nuclear staining). The slide was then finally washed with tap water, dehydrated in ethanol and cleared in xylene before visualisation under the microscope.

Semi-quantitative estimates of cell counts were made in × 200 magnification medium power fields. Tissue specimens were classified as having 0 = no cells; 1 = a few cells; 2 = moderate cells or 3 = abundant cells [7]. Using the labelled polymer immune-peroxidase method, a brown cellular stain indicated a positive stain for an inflammatory cell. On the assumption that inflammatory sites might be variably located within the disc tissue, the slide demonstrating the most evidence of inflammation granulation infiltrate was selected from which cellular evidence of inflammation would be determined. Histologically confirmed inflammation was defined as at least moderate cells of any type in a specimen. Cell counts were done by an independent anatomical pathologist with 23-years of specialist experience. The pathologist was blinded to any clinical or demographic information on the origin of the disc material.

### Potential clinical features of inflammation

All patients having discectomy underwent a comprehensive and standardised assessment prior to their surgery upon admission to hospital blinded to any tests regarding histologically confirmed inflammation. Clinical assessment items included sociodemographic factors, low back pain-related subjective examination (measuring symptom duration, location and nature of symptoms, pain drawing, aggravating and easing factors, and history of symptoms) [10], low back pain-related physical examination (measuring active movement testing, straight leg raise, crossed straight leg raise, provocative sacro-iliac joint testing, lower limb neurological examination, response to mechanical loading strategies and lumbar palpation) and psychosocial risk factors comprising the Örebro Musculoskeletal Pain Questionnaire [28] and non-organic signs [47]. The clinical assessment had acceptable evidence of reliability and validity [3, 10, 12, 16, 19, 50].

Self-administered standardized outcome measures were completed comprising valid and reliable measures of activity limitation (Oswestry Disability Index) [13, 31] and a series of visual analogue scales [23, 29] as a measure of overall symptoms, back symptoms and leg symptoms.

Magnetic resonance imaging (MRI) scans were reported by a study radiologist, blinded to the baseline clinical assessment/patient outcomes, who assessed the patient’s MRI scan using a reliable and valid protocol [9, 34].

Based on previous diagnostic accuracy studies in LBP [20, 40, 44], clinical features are more likely to be predictive when multiple features are combined into a composite score. Research suggests that certain subjective examination items (age at onset < 40 years, insidious onset, improvement with exercise, no improvement with rest and pain at night associated with improvement upon getting out of bed) are indicative of IBP in people with spondyloarthropathy [49]. However, the mechanisms underpinning IBP are substantially different to LBP with an inflammatory component due to DHR. As such the clinical features for IBP were modified for the selected sample based on expert opinion [21, 48] and evidence of prognostic ability [11] to form a composite clinical inflammation score as a potential predictor. A positive on this score was at least three of: constant symptoms, morning pain/stiffness greater than 60-min, short walking not easing symptoms and significant night symptoms (waking most/every night, **plus** waking is not due to movement in bed and/or unable to return to sleep without sitting up, getting out of bed or taking medication).

Potential predictors evaluated therefore included all items from the clinical assessment, outcome measures, MRI scans and the composite clinical inflammation score.

### Statistical analysis

All potential predictors of histologically confirmed inflammation were assessed for multicollinearity, which was considered likely if correlations between factors were > 0.8 [6]. For univariate analysis, each potential predictor was tested for its association with histologically confirmed inflammation via chi-square testing (Fisher’s Exact test when cell values less than five were present), Spearman’s rank order correlation, or Pearson’s correlation for nominal, ordinal and continuous data respectively. Significant univariate predictors (*p* < 0.05) then progressed to multivariate logistic regression analysis. To avoid overfitting models with our relatively small sample size, we applied a maximum limit of 8 factors to progress to multivariate analysis based on the guideline of one factor for every five to ten patients [46]. In the multivariate stage, a Wald backwards stepwise approach was utilised. It was planned to report the multivariate model containing all significant univariate predictors (Model 1), the final model containing only independently significant predictors after backward deletion (Model 2), and a third model displaying the best balance of parsimony and performance (e.g. highest sensitivity and specificity with the fewest predictors). All analyses were undertaken using SPSS 22 and Microsoft Excel.

## Results

Histology was undertaken on the disc tissue of 43 patients. Three of these patients (all negative for inflammation) had no baseline data available so they were excluded from the study. The clinical characteristics of the final sample (*n* = 40) are outlined in Table 1. All herniated disc specimens were from lower lumbar discs (L3/4 to L5/S1). Three female control patients, aged 16, 18 and 28-years provided a specimen from a single lower lumbar disc (L3/4 to L5/S1).

**Table 1:** Clinical characteristics of patients (*n* = 40)

Characteristic	Mean (SD) or N (%)
Age (years)	43.5 (14.7)
Gender (Male)	31 (77.5%)
Smoker	8 (20.0%)
Compensation claim	10 (25.0%)
Back pain (VAS /10)	5.3 (2.1)
Leg pain (VAS /10)	6.3 (2.3)
Pain or paraesthesia below knee	37 (92.5%)
First episode	17 (42.5%)
Duration of symptoms (current episode)	
1 week – 1 month	8 (20.0%)
2–3 months	8 (20.0%)
4–6 months	13 (32.5%)
7–12 months	2 (5.0%)
> 12 months	9 (22.5%)
Activity limitation (Oswestry)	40.1 (15.8)
Örebro score	115.7 (22.8)
MRI findings	
Herniation type	
Bulge or normal	1 (2.5%)
Protrusion	16 (40.0%)
Extrusion	21 (52.5%)
Sequestration	1 (2.5%)
Nerve root involvement	
None	0 (0.0%)
Contact	2 (5.0%)
Displacement	6 (15%)
Compression	31 (77.5%)
Annular tear	
None	4 (10.3%)
Mild tear	23 (59.0%)
Severe tear	12 (30.8%)
Physical examination findings:	
Ipsilateral SLR: mean (SD) degrees	50.2 (17.8)
Contralateral SLR: mean (SD) degrees	69.5 (15.8)
Neurological deficit (affected side)	
Reflex deficit	28 (70.0%)
Myotomal deficit	16 (40.0%)
Dermatomal deficit	31 (77.5%)
At least one neurological deficit	38 (95.0%)

*SD* Standard deviation, *N* Number of patients, *%* Percentage, *VAS* Visual analogue scale, *SLR* Straight Leg Raise, *MRI* Magnetic resonance imaging, *Örebro* Örebro Musculoskeletal Pain Questionnaire

**Fig. 1:**
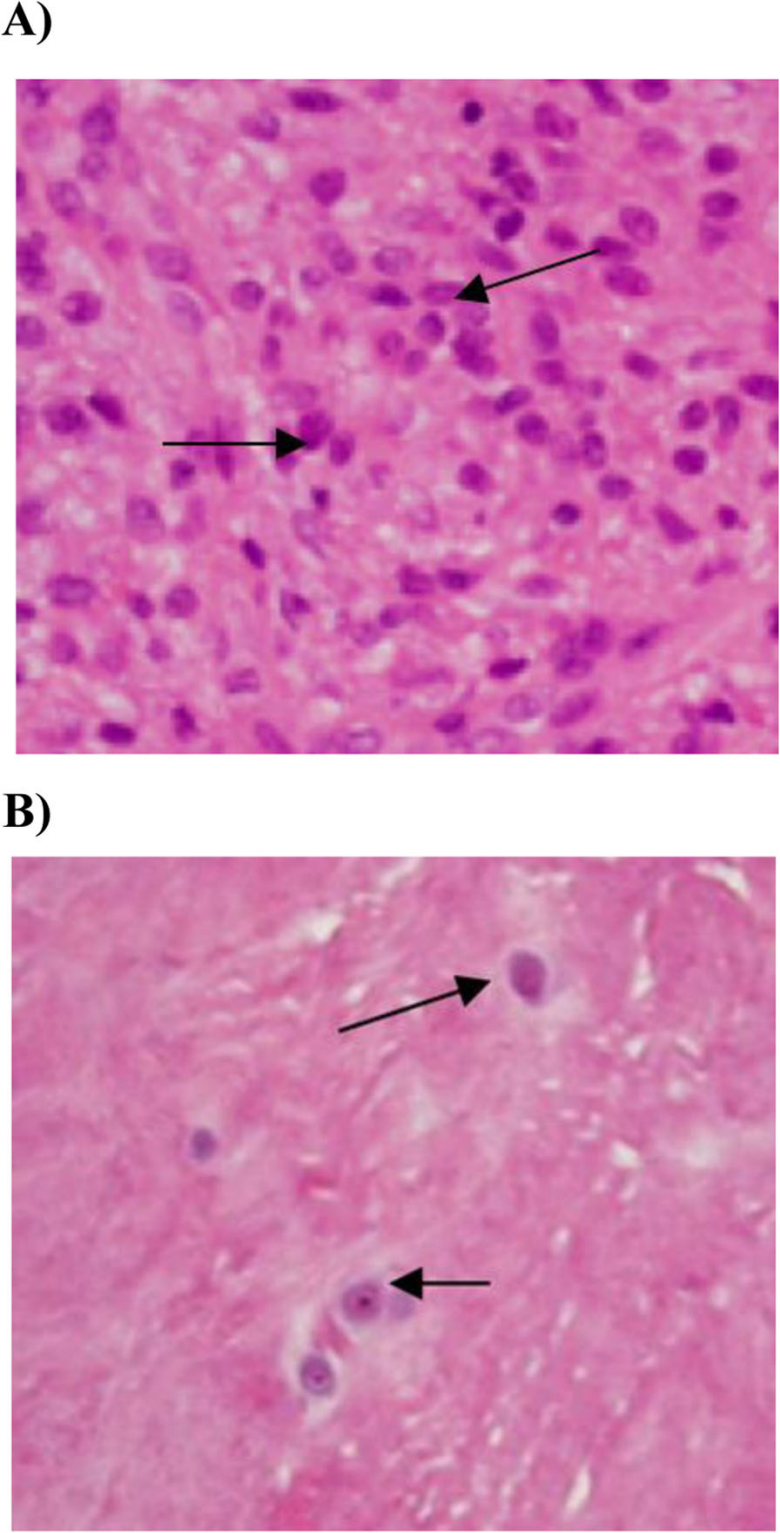
Hematoxylin and eosin stained sections. Original magnification × 200. Herniated disc sample showing inflammatory granulation tissue infiltration (**a**), Control disc sample showing chondrocyte nuclei and no evidence of inflammatory cell infiltration (**b**)

**Fig. 2:**
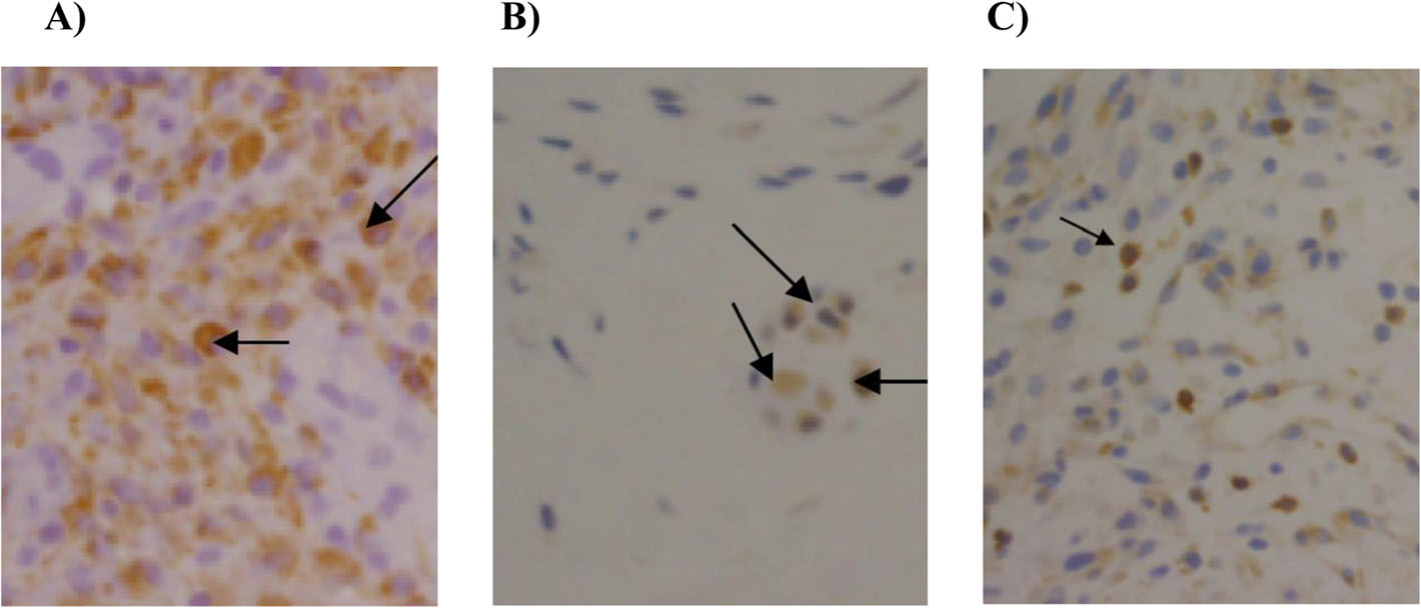
Immunohistochemical staining of herniated disc tissue sections with inflammatory cells brown with a blue nucleus. Original magnification × 200. CD68-positive macrophages (3 = abundant) (**a**), CD20-positive T lymphocytes (1 = a few cells) (**b**), CD3-positive B lymphocytes (1 = a few cells) (C)

**Fig. 3:**
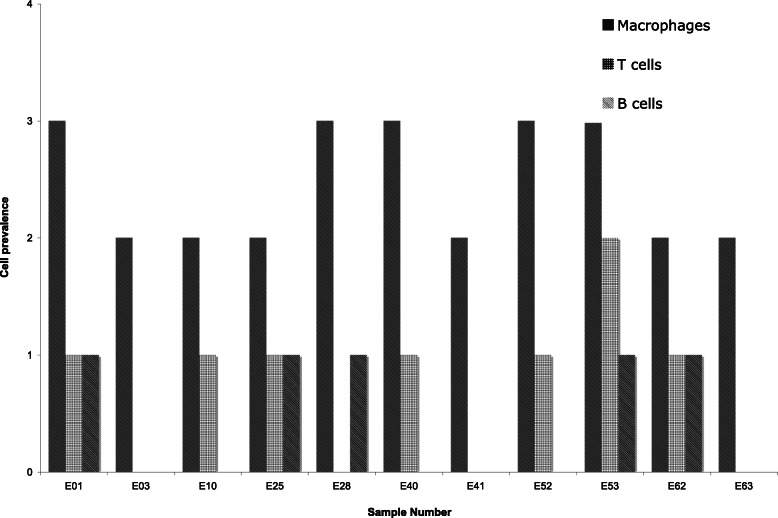
Relative prevalence of inflammatory cells in immunohistochemical staining of herniated disc tissue sections. Cell prevalence: 0 = no cells, 1 = few cells, 2 = moderate cells, 3 = abundant cells

Of the 40 DHR patients in the study, 11 (28%) had at least moderate histological evidence of inflammation and were scored as positive for inflammation. All of these specimens demonstrated typical haematoxylin and eosin stained features of granulation tissue, which was predominately composed of infiltrating large mononuclear cells (Fig. 1). Immunohistochemical staining with monoclonal antibodies showed moderate to abundant infiltration of CD68-positive macrophages in all specimens (Fig. 2). In contrast, CD3-positive T lymphocytes and CD20-positive B lymphocytes were not detected in abundance, demonstrating few to moderate cell counts. The relative prevalence of inflammatory cells for each of the specimens is displayed in Fig. 3.

Hematoxylin and eosin staining of control disc tissue revealed no evidence of infiltration of inflammatory granulation tissue (Fig. 1).

### Clinical features predicting histologically confirmed inflammation

On univariate analysis, six clinical features showed a statistically significant association with histologically confirmed inflammation (Additional file 1). Additional files 2 and 3 provide the results for all univariate features analysed. Back pain < 5/10 was the variable with the best individual predictive value for histologically confirmed inflammation, with a sensitivity of 72.7%, a specificity of 82.8%, and correct prediction of the presence or absence of inflammation in 80.0% of patients. The six significant features on univariate analysis progressed to multivariate logistic regression analysis to develop clinical models predictive of histologically confirmed inflammation.

Tables 2 and 3 show the multivariate models. After Wald backward elimination of the six significant univariate features, two features remained as significant independent predictors of inflammation in the final model (clinical inflammation score of 3 or more, and back pain < 5/10 on the VAS). A model containing four features was found to have the best balance of performance and parsimony. This model achieved a sensitivity of 90.9%, a specificity of 92.9% and predictive accuracy of 92.3% for the presence or absence of inflammation, with positive and negative likelihood ratios of 12.7 and 0.1 respectively (Table 3).

**Table 2:** **Multivariate model**s **for predicting histologically confirmed inflammation**

	B (intercept)	***p***-value	Exp(B) (odds ratio)	Lower 95%CI for Exp(B)	Upper 95%CI for Exp(B)
**Model 1: (all significant univariate factors included)**					
Clinical inflammation score >/=3	2.7	0.05	15.2	1.0	233.6
Back pain < 5/10	2.2	0.19	9.1	0.3	258.0
Can sit with a firm backrest > 30 min	0.2	0.90	1.2	0.1	24.3
Worse the next day after injury	1.2	0.35	3.2	0.3	36.2
Flexion range of motion 0–30°	1.1	0.37	3.1	0.3	38.2
MRI - disc extrusion	1.0	0.47	2.7	0.2	40.4
Intercept	−4.9	0.00	0.0		
**Model 2: (Final model with only significant predictors remaining)**					
Clinical inflammation score >/=3	2.8	0.03	16.5	1.4	195.3
Back pain < 5/10	3.1	0.01	23.1	2.4	224.6
Constant	−3.1	0.00	0.0		
**Model 3: Best balance of model parsimony and performance**					
Clinical inflammation score >/=3	2.6	0.04	12.8	1.1	154.7
Back pain < 5/10	2.8	0.02	16.4	1.6	172.7
Worse the next day after injury	1.4	0.25	3.9	0.4	39.2
Flexion range of motion 0–30°	1.4	0.24	4.0	0.4	40.9
Constant	−4.8	0.00	0.0		

*B* Coefficient for the constant (intercept), *Exp(B)* Odds ratio, *CI* Confidence interval for the odds ratio, *MRI* Magnetic resonance imaging, *Constant* Constant symptoms

**Table 3:** Performance of multivariate models for predicting histologically confirmed inflammation

		Histology negative for inflammation		Histology positive for inflammation								
Model	N	Predicted negative for inflammation	Predicted positive for inflammation	Predicted negative for inflammation	Predicted positive for inflammation	Sensitivity	Specificity	% correctly predicted	LR+	LR-	Diagnostic Odds Ratio	R-square (Cox & Snell)
Model 1	37	24	2	1	10	90.9%	92.3%	91.9%	11.8	0.1	120.0	0.42
Model 2	40	24	5	3	8	72.7%	82.8%	80.0%	4.2	0.3	12.8	0.36
Model 3	39	26	2	1	10	90.9%	92.9%	92.3%	12.7	0.1	130.0	0.42

*N* Number of samples, *%* Percentage, *LR+* Positive likelihood ratio, *LR-* Negative likelihood ratio, *R-square* Coefficient of determination – the proportion of variance in the presence or absence of inflammation in the disc that is explained by the predictive model

## Discussion

This results of this study show that the combined clinical features of back pain < 5/10, symptoms being worse the next day after injury, a lumbar flexion range of motion between 0 and 30° and the composite inflammation score (at least 3 of constant symptoms, morning pain/stiffness greater than 60-min, short walking not easing symptoms and significant night symptoms) predicted the presence or absence of histologically confirmed inflammation in disc specimens removed from patients undergoing surgical discectomy for DHR. The validity of these findings was strengthened by the fact that there was no histological evidence of inflammation in three control specimens from three patients with no current or recent LBP. This appears to be the first study to evaluate the diagnostic accuracy of clinical features in predicting histological or other markers of inflammation in patients with lumbar DHR. Identifying clinical features of inflammation in LBP has the potential of allowing more targeted anti-inflammatory treatment in future RCTs and in clinical practice.

The data show high levels of diagnostic accuracy when compared with other tests evaluated in the LBP literature to date. Systematic reviews on patients with zygapophyseal joint dysfunction [30], lumbar radiculopathy [41] and serious pathologies causing back pain [44] show limited diagnostic accuracy of clinical features. There is some evidence of diagnostic accuracy [20] for a combination of clinical tests in sacro-iliac joint dysfunction (positive likelihood ratio of 3.2); and centralisation for discogenic pain (positive likelihood ratio of 2.8) however these parameters are lower than those reported in our study, particularly for our models combining multiple clinical features. Several of the predictive clinical features are consistent with those accepted as indicative of inflammatory processes in patients with spondyloarthropathy [2, 24, 39, 42] despite the putative mechanisms being different to LBP and DHR. They are also consistent with an expert panel study on the clinical features of LBP in association with inflammation and in the absence of IBP [48].

## Limitations

This study had a relatively small sample size and further validation of the multivariate model on a larger sample size is required. The identified clinical features are only applicable to people with DHR limiting generalisability. However, it is plausible that the mechanisms underpinning these clinical features could also apply to other types of LBP and potentially other musculoskeletal conditions. The method of deriving the composite clinical inflammation score has not been previously validated but is consistent with other clinical tests for LBP where combinations of tests are considered more likely to be informative than single tests alone [20, 40, 44]. The composite score was also derived from the literature on IBP, clinical experts in the area of LBP [48] and evidence of prognostic ability [11]. Only three relatively young female control patients were tested for histological evidence of inflammation due to feasibility issues in obtaining age matched subjects however the negative results in these asymptomatic cases are consistent with data from other studies [15, 18, 45].

## Conclusion

This study involving patients with lumbar disc herniation and associated radiculopathy showed that a combination of clinical features predicted the presence or absence of histologically confirmed inflammation. Further research is required to externally validate these findings in different types of LBP and other musculoskeletal conditions. The identified clinical features of inflammation have the potential to allow targeted anti-inflammatory treatment in future RCTs and in clinical practice.

## Supplementary information

**Additional file 1.** All significant univariate predictors of histologically confirmed inflammation.

**Additional file 2.** Univariate analysis of dichotomous variables for predicting histologically confirmed inflammation.

**Additional file 3.** Univariate analysis of ordinal, continuous, and multi-nominal variables for predicting histologically confirmed inflammation.

## Data Availability

The data generated and//or analysed during the current study are not publicly available but are available from the corresponding author on reasonable request.

## Abbreviations

DHR: Disc herniation and associated radiculopathy
RCT: Randomised controlled trail
LBP: Low back pain
NSAID: Non-steroidal anti-inflammatory drugs
IBP: Inflammatory back pain
MRI: Magnetic resonance imaging
PBS: Phosphate buffered saline
VAS: Visual analogue scale
SLR: Straight leg raise
N: Number of samples
LR+: Likelihood ratio positive
LR-: Likelihood ratio negative

## Footnotes

1 DAKO, Copenhagen, Denmark

2 Vision Biosystems, Melbourne, Australia

3 DAKO, Copenhagen, Denmark

4 DAKO, Copenhagen, Denmark

5 DAKO, Copenhagen, Denmark

6 DAKO, Copenhagen, Denmark
