# Supplementary material for: Clinical features as predictors of histologically confirmed inflammation in patients with lumbar disc herniation with associated radiculopathy
Source: BMC Musculoskelet Disord. 2020 Aug 21;21:567. doi: 10.1186/s12891-020-03590-x (PMC7442978; doi:10.1186/s12891-020-03590-x)
Supplement: Supplementary file 1 — Additional file 1. All significant univariate predictors of histologically confirmed inflammation. [file 12891_2020_3590_MOESM1_ESM.docx]

**Additional file 1: All significant univariate predictors of histologically confirmed inflammation**

|  |  | **Histology positive for inflammation** | | **Histology negative for inflammation** | |  |  |  |  |  |  |  |
| --- | --- | --- | --- | --- | --- | --- | --- | --- | --- | --- | --- | --- |
| **Predictor** | **N** | **Predictor "no"** | **Predictor "yes"** | **Predictor "no"** | **Predictor "yes"** | **P-value** | **Sensitivity** | **Specificity** | **% correctly predicted** | **+ve**  **LR** | **-ve**  **LR** | **Diagnostic Odds Ratio** |
| Back pain <5/10 | 40 | 24 | 5 | 3 | 8 | <.01 | 72.7% | 82.8% | 80.0% | 4.2 | 0.3 | 12.8 |
| Can sit with a firm backrest >30 minutes | 39 | 22 | 6 | 4 | 7 | 0.02 | 63.6% | 78.6% | 74.4% | 3.0 | 0.5 | 6.4 |
| Composite clinical inflammation score >/=3 | 40 | 26 | 3 | 6 | 5 | 0.03 | 45.5% | 89.7% | 77.5% | 4.4 | 0.6 | 7.2 |
| Worse next day after injury | 40 | 18 | 11 | 2 | 9 | 0.03 | 81.8% | 62.1% | 67.5% | 2.2 | 0.3 | 7.4 |
| Flexion range of motion 0-30° | 39 | 16 | 12 | 2 | 9 | 0.04 | 81.8% | 57.1% | 64.1% | 1.9 | 0.3 | 6.0 |
| MRI - disc extrusion | 39 | 16 | 12 | 2 | 9 | 0.04 | 81.8% | 57.1% | 64.1% | 1.9 | 0.3 | 6.0 |
| >3 of the above 6 features | 40 | 27 | 2 | 2 | 9 | <.01 | 81.8% | 93.1% | 90.0% | 11.9 | 0.2 | 60.8 |
| >4 of the above 6 features | 40 | 28 | 1 | 4 | 7 | <.01 | 63.6% | 96.6% | 87.5% | 18.5 | 0.4 | 49.0 |

LR=likelihood ratio, MRI=magnetic resonance imaging
