# Supplementary material for: Clinical features as predictors of histologically confirmed inflammation in patients with lumbar disc herniation with associated radiculopathy
Source: BMC Musculoskelet Disord. 2020 Aug 21;21:567. doi: 10.1186/s12891-020-03590-x (PMC7442978; doi:10.1186/s12891-020-03590-x)
Supplement: Supplementary file 2 — Additional file 2. Univariate analysis of dichotomous variables for predicting histologically confirmed inflammation. [file 12891_2020_3590_MOESM2_ESM.docx]

**Additional file 2: Univariate analysis of dichotomous variables for predicting histologically confirmed inflammation**

|  |  | Histology positive for inflammation | | Histology negative for inflammation | |  |  |  |  |  |  |  |  |  |
| --- | --- | --- | --- | --- | --- | --- | --- | --- | --- | --- | --- | --- | --- | --- |
| **Predictor** | **N** | **Predictor “no”** | **Predictor “yes”** | **Predictor “no”** | **Predictor “yes”** | **P-value** | **Sensitivity** | **Specificity** | **Positive predictive value** | **Negative predictive value** | **% correctly predicted** | **LR+** | **LR-** | **Diagnostic Odds Ratio** |
| Back pain < 5/10 | 40 | 24 | 5 | 3 | 8 | 0.002 | 72.7% | 82.8% | 61.5% | 88.9% | 80.0% | 4.2 | 0.3 | 12.8 |
| Can sit with a firm backrest >30 minutes | 39 | 22 | 6 | 4 | 7 | 0.022 | 63.6% | 78.6% | 53.8% | 84.6% | 74.4% | 3.0 | 0.5 | 6.4 |
| Clinical inflammation score >3 | 40 | 26 | 3 | 6 | 5 | 0.025 | 45.5% | 89.7% | 62.5% | 81.3% | 77.5% | 4.4 | 0.6 | 7.2 |
| Worse the next day after injury | 40 | 18 | 11 | 2 | 9 | 0.031 | 81.8% | 62.1% | 45.0% | 90.0% | 67.5% | 2.2 | 0.3 | 7.4 |
| Flexion range-of-motion 0-30 degrees | 39 | 16 | 12 | 2 | 9 | 0.037 | 81.8% | 57.1% | 42.9% | 88.9% | 64.1% | 1.9 | 0.3 | 6.0 |
| MRI - disc extrusion | 39 | 16 | 12 | 2 | 9 | 0.037 | 81.8% | 57.1% | 42.9% | 88.9% | 64.1% | 1.9 | 0.3 | 6.0 |
| *>3 of the above 6 features* | 40 | 27 | 2 | 2 | 9 | <.001 | 81.8% | 93.1% | 81.8% | 93.1% | 90.0% | 11.9 | 0.2 | 60.8 |
| *>4 of the above 6 features* | 40 | 28 | 1 | 4 | 7 | <.001 | 63.6% | 96.6% | 87.5% | 87.5% | 87.5% | 18.5 | 0.4 | 49.0 |
| Slouched sitting on a couch eases | 40 | 26 | 3 | 7 | 4 | 0.075 | 36.4% | 89.7% | 57.1% | 78.8% | 75.0% | 3.5 | 0.7 | 5.0 |
| MRI - Nerve root contact - Compression | 39 | 8 | 20 | 0 | 11 | 0.078 | 100.0% | 28.6% | 35.5% | 100.0% | 48.7% | 1.4 | 0.0 | N/A |
| Symptoms half the time or less (ie. episodic) | 40 | 19 | 7 | 4 | 6 | 0.119 | 60.0% | 73.1% | 46.2% | 82.6% | 69.4% | 2.2 | 0.5 | 4.1 |
| Altered reflex on ipsilateral side | 40 | 11 | 18 | 1 | 10 | 0.124 | 90.9% | 37.9% | 35.7% | 91.7% | 52.5% | 1.5 | 0.2 | 6.1 |
| Short walk or movement eases | 40 | 6 | 23 | 5 | 6 | 0.137 | 54.5% | 20.7% | 20.7% | 54.5% | 30.0% | 0.7 | 2.2 | 0.3 |
| Able to stand for 15 mins or more | 40 | 23 | 6 | 6 | 5 | 0.137 | 45.5% | 79.3% | 45.5% | 79.3% | 70.0% | 2.2 | 0.7 | 3.2 |
| Contralateral SLR movement <60° | 40 | 25 | 4 | 7 | 4 | 0.182 | 36.4% | 86.2% | 50.0% | 78.1% | 72.5% | 2.6 | 0.7 | 3.6 |
| Altered dermatome on ipsilateral side | 40 | 5 | 24 | 4 | 7 | 0.227 | 63.6% | 17.2% | 22.6% | 55.6% | 30.0% | 0.8 | 2.1 | 0.4 |
| Örebro score >105 | 40 | 9 | 20 | 1 | 10 | 0.233 | 90.9% | 31.0% | 33.3% | 90.0% | 47.5% | 1.3 | 0.3 | 4.5 |
| Waking at night - due to pain without moving | 40 | 22 | 7 | 6 | 5 | 0.254 | 45.5% | 75.9% | 41.7% | 78.6% | 67.5% | 1.9 | 0.7 | 2.6 |
| Contralateral SLR reproduces pain | 40 | 22 | 7 | 6 | 5 | 0.254 | 45.5% | 75.9% | 41.7% | 78.6% | 67.5% | 1.9 | 0.7 | 2.6 |
| Coughing "fairly difficult" or worse | 40 | 9 | 20 | 6 | 5 | 0.273 | 45.5% | 31.0% | 20.0% | 60.0% | 35.0% | 0.7 | 1.8 | 0.4 |
| Contralateral SLR sensitisation positive | 40 | 20 | 9 | 5 | 6 | 0.273 | 54.5% | 69.0% | 40.0% | 80.0% | 65.0% | 1.8 | 0.7 | 2.7 |
| Sit to stand at least "fairly severe" | 40 | 17 | 12 | 4 | 7 | 0.293 | 63.6% | 58.6% | 36.8% | 81.0% | 60.0% | 1.5 | 0.6 | 2.5 |
| Morning stiffness - any | 40 | 10 | 19 | 6 | 5 | 0.295 | 45.5% | 34.5% | 20.8% | 62.5% | 37.5% | 0.7 | 1.6 | 0.4 |
| Duration of current episode </= 3 months | 40 | 19 | 10 | 5 | 6 | 0.295 | 54.5% | 65.5% | 37.5% | 79.2% | 62.5% | 1.6 | 0.7 | 2.3 |
| Repeated EIL results in centralisation | 40 | 21 | 8 | 10 | 1 | 0.399 | 9.1% | 72.4% | 11.1% | 67.7% | 55.0% | 0.3 | 1.3 | 0.3 |
| Any centralisation with repeated EIL or sustained EIL | 40 | 21 | 8 | 10 | 1 | 0.399 | 9.1% | 72.4% | 11.1% | 67.7% | 55.0% | 0.3 | 1.3 | 0.3 |
| Trouble getting to sleep every night | 40 | 22 | 7 | 10 | 1 | 0.405 | 9.1% | 75.9% | 12.5% | 68.8% | 57.5% | 0.4 | 1.2 | 0.3 |
| Smoker | 40 | 22 | 7 | 10 | 1 | 0.405 | 9.1% | 75.9% | 12.5% | 68.8% | 57.5% | 0.4 | 1.2 | 0.3 |
| Can sit slouched >30 minutes | 40 | 23 | 6 | 7 | 4 | 0.418 | 36.4% | 79.3% | 40.0% | 76.7% | 67.5% | 1.8 | 0.8 | 2.2 |
| Constant pain | 40 | 10 | 19 | 2 | 9 | 0.451 | 81.8% | 34.5% | 32.1% | 83.3% | 47.5% | 1.2 | 0.5 | 2.4 |
| Pain is the same at all times of the day | 40 | 21 | 8 | 6 | 5 | 0.451 | 45.5% | 72.4% | 38.5% | 77.8% | 65.0% | 1.6 | 0.8 | 2.2 |
| Crook lying eases | 40 | 7 | 22 | 4 | 7 | 0.455 | 63.6% | 24.1% | 24.1% | 63.6% | 35.0% | 0.8 | 1.5 | 0.6 |
| Repeated EIL - no change | 40 | 22 | 7 | 7 | 4 | 0.455 | 36.4% | 75.9% | 36.4% | 75.9% | 65.0% | 1.5 | 0.8 | 1.8 |
| Short walk eases | 40 | 12 | 17 | 6 | 4 | 0.465 | 40.0% | 41.4% | 19.0% | 66.7% | 41.0% | 0.7 | 1.5 | 0.5 |
| Movement eases | 40 | 9 | 20 | 5 | 6 | 0.469 | 54.5% | 31.0% | 23.1% | 64.3% | 37.5% | 0.8 | 1.5 | 0.5 |
| Worst pain location is lower leg | 40 | 20 | 9 | 6 | 5 | 0.469 | 45.5% | 69.0% | 35.7% | 76.9% | 62.5% | 1.5 | 0.8 | 1.9 |
| Lying prone eases | 40 | 9 | 20 | 5 | 6 | 0.469 | 54.5% | 31.0% | 23.1% | 64.3% | 37.5% | 0.8 | 1.5 | 0.5 |
| Altered myotome on ipsilateral side | 40 | 16 | 13 | 8 | 3 | 0.473 | 27.3% | 55.2% | 18.8% | 66.7% | 47.5% | 0.6 | 1.3 | 0.5 |
| Can lie prone >/= 30 minutes | 40 | 18 | 11 | 5 | 6 | 0.477 | 54.5% | 62.1% | 35.3% | 78.3% | 60.0% | 1.4 | 0.7 | 2.0 |
| Mechanism of injury - sudden onset with heavy work | 40 | 18 | 11 | 5 | 6 | 0.477 | 54.5% | 62.1% | 35.3% | 78.3% | 60.0% | 1.4 | 0.7 | 2.0 |
| Lying on painful side eases | 39 | 26 | 2 | 9 | 2 | 0.562 | 18.2% | 92.9% | 50.0% | 74.3% | 71.8% | 2.5 | 0.9 | 2.9 |
| Female gender | 40 | 23 | 6 | 8 | 3 | 0.686 | 27.3% | 79.3% | 33.3% | 74.2% | 65.0% | 1.3 | 0.9 | 1.4 |
| Below knee pain | 40 | 6 | 23 | 3 | 8 | 0.686 | 72.7% | 20.7% | 25.8% | 66.7% | 35.0% | 0.9 | 1.3 | 0.7 |
| Positive score on night symptoms | 40 | 20 | 9 | 9 | 2 | 0.694 | 18.2% | 69.0% | 18.2% | 69.0% | 55.0% | 0.6 | 1.2 | 0.5 |
| Get back to sleep - nothing helps or has to take medication | 40 | 20 | 9 | 9 | 2 | 0.694 | 18.2% | 69.0% | 18.2% | 69.0% | 55.0% | 0.6 | 1.2 | 0.5 |
| NSAIDs help | 40 | 21 | 8 | 9 | 2 | 0.696 | 18.2% | 72.4% | 20.0% | 70.0% | 57.5% | 0.7 | 1.1 | 0.6 |
| Compensation claim present | 40 | 21 | 8 | 9 | 2 | 0.696 | 18.2% | 72.4% | 20.0% | 70.0% | 57.5% | 0.7 | 1.1 | 0.6 |
| Foot drop present | 40 | 21 | 8 | 9 | 2 | 0.696 | 18.2% | 72.4% | 20.0% | 70.0% | 57.5% | 0.7 | 1.1 | 0.6 |
| Ipsilateral neurological signs x 3 | 40 | 21 | 8 | 9 | 2 | 0.696 | 18.2% | 72.4% | 20.0% | 70.0% | 57.5% | 0.7 | 1.1 | 0.6 |
| Below knee parasthesia | 40 | 8 | 21 | 4 | 7 | 0.704 | 63.6% | 27.6% | 25.0% | 66.7% | 37.5% | 0.9 | 1.3 | 0.7 |
| Lying on less painful side eases | 36 | 11 | 15 | 3 | 7 | 0.706 | 70.0% | 42.3% | 31.8% | 78.6% | 50.0% | 1.2 | 0.7 | 1.7 |
| MRI – severe annular tear - | 39 | 20 | 8 | 7 | 4 | 0.709 | 36.4% | 71.4% | 33.3% | 74.1% | 61.5% | 1.3 | 0.9 | 1.4 |
| Static EIL produces radicular pain | 40 | 20 | 8 | 7 | 4 | 0.709 | 36.4% | 71.4% | 33.3% | 74.1% | 61.5% | 1.3 | 0.9 | 1.4 |
| Waking most nights or every night | 40 | 18 | 11 | 8 | 3 | 0.715 | 27.3% | 62.1% | 21.4% | 69.2% | 52.5% | 0.7 | 1.2 | 0.6 |
| Morning stiffness >60 minutes | 40 | 19 | 10 | 6 | 5 | 0.716 | 45.5% | 65.5% | 33.3% | 76.0% | 60.0% | 1.3 | 0.8 | 1.6 |
| Sitting firm backrest eases | 40 | 19 | 10 | 6 | 5 | 0.716 | 45.5% | 65.5% | 33.3% | 76.0% | 60.0% | 1.3 | 0.8 | 1.6 |
| Repeated EIL - peripheralisation | 40 | 16 | 13 | 5 | 6 | 0.727 | 54.5% | 55.2% | 31.6% | 76.2% | 55.0% | 1.2 | 0.8 | 1.5 |
| Unable to get back to sleep without sitting up, GOOB or taking medication, or can’t return to sleep at all | 40 | 20 | 9 | 7 | 4 | 1 | 36.4% | 69.0% | 30.8% | 74.1% | 60.0% | 1.2 | 0.9 | 1.3 |
| Getting back to sleep - nothing helps | 40 | 28 | 1 | 11 | 0 | 1 | 0.0% | 96.6% | 0.0% | 71.8% | 70.0% | 0.0 | 1.0 | 0.0 |
| Getting back to sleep – has to GOOB | 40 | 28 | 1 | 11 | 0 | 1 | 0.0% | 96.6% | 0.0% | 71.8% | 70.0% | 0.0 | 1.0 | 0.0 |
| Getting back to sleep – has to take medication | 40 | 22 | 7 | 9 | 2 | 1 | 18.2% | 75.9% | 22.2% | 71.0% | 60.0% | 0.8 | 1.1 | 0.7 |
| Below knee pain or parasthesia | 40 | 2 | 27 | 1 | 10 | 1 | 90.9% | 6.9% | 27.0% | 66.7% | 30.0% | 1.0 | 1.3 | 0.7 |
| Standing eases | 40 | 24 | 5 | 9 | 2 | 1 | 18.2% | 82.8% | 28.6% | 72.7% | 65.0% | 1.1 | 1.0 | 1.1 |
| First episode of pain | 40 | 17 | 12 | 6 | 5 | 1 | 45.5% | 58.6% | 29.4% | 73.9% | 55.0% | 1.1 | 0.9 | 1.2 |
| Ever had a manual handling job | 40 | 12 | 17 | 4 | 7 | 1 | 63.6% | 41.4% | 29.2% | 75.0% | 47.5% | 1.1 | 0.9 | 1.2 |
| Örebro >130 | 40 | 21 | 8 | 8 | 3 | 1 | 27.3% | 72.4% | 27.3% | 72.4% | 60.0% | 1.0 | 1.0 | 1.0 |
| Ipsilateral SLR reproduces pain | 40 | 5 | 24 | 1 | 10 | 1 | 90.9% | 17.2% | 29.4% | 83.3% | 37.5% | 1.1 | 0.5 | 2.1 |
| Ipsilateral SLR sensitisation positive | 40 | 6 | 23 | 2 | 9 | 1 | 81.8% | 20.7% | 28.1% | 75.0% | 37.5% | 1.0 | 0.9 | 1.2 |
| Any neurological signs | 40 | 2 | 27 | 0 | 11 | 1 | 100.0% | 6.9% | 28.9% | 100.0% | 32.5% | 1.1 | 0.0 | N/A |
| Ipsilateral neurological signs >0 | 40 | 2 | 27 | 0 | 11 | 1 | 100.0% | 6.9% | 28.9% | 100.0% | 32.5% | 1.1 | 0.0 | N/A |
| Ipsilateral neurological signs >1 | 40 | 9 | 20 | 4 | 7 | 1 | 63.6% | 31.0% | 25.9% | 69.2% | 40.0% | 0.9 | 1.2 | 0.8 |
| Focal palpation findings | 40 | 15 | 12 | 5 | 5 | 1 | 50.0% | 55.6% | 29.4% | 75.0% | 54.1% | 1.1 | 0.9 | 1.3 |

MRI=magnetic resonance imaging, EIL=extension in lying, NSAIDs=non-steroidal anti-inflammatory medication, GOOB=getting out of bed, SLR=straight leg raise
