# Supplementary material for: Clinical features as predictors of histologically confirmed inflammation in patients with lumbar disc herniation with associated radiculopathy
Source: BMC Musculoskelet Disord. 2020 Aug 21;21:567. doi: 10.1186/s12891-020-03590-x (PMC7442978; doi:10.1186/s12891-020-03590-x)
Supplement: Supplementary file 3 — Additional file 3. Univariate analysis of ordinal, continuous, and multi-nominal variables for predicting histologically confirmed inflammation. [file 12891_2020_3590_MOESM3_ESM.docx]

**Additional file 3: Univariate analysis of ordinal, continuous, and multi-nominal variables for predicting histologically confirmed inflammation**

| Predictor | N | Type of variable | P-value |
| --- | --- | --- | --- |
| Sitting straight with a firm backrest | 40 | Ordinal | 0.025 |
| Sit-to-stand | 40 | Ordinal | 0.192 |
| Slouched sitting on a couch | 40 | Ordinal | 0.22 |
| Coughing or sneezing | 40 | Ordinal | 0.232 |
| Clinical inflammation score | 40 | Ordinal | 0.348 |
| Duration of current episode | 40 | Ordinal | 0.529 |
| Walking | 39 | Ordinal | 0.577 |
| Night symptoms (total number) | 40 | Ordinal | 0.63 |
| Prone lying | 40 | Ordinal | 0.672 |
| Standing still | 40 | Ordinal | 0.682 |
| Night symptoms (total number): includes can’t GBTS | 40 | Ordinal | 0.684 |
| Number of ipsilateral neuro signs | 40 | Ordinal | 0.693 |
| Waddell’s non-organic score | 40 | Ordinal | 0.917 |
| SIJ score | 40 | Ordinal | 0.963 |
| Back pain VAS / 10 | 40 | Continuous | 0.041 |
| Flexion ROM | 40 | Continuous | 0.145 |
| Leg pain - VAS | 36 | Continuous | 0.232 |
| Orebro score | 40 | Continuous | 0.256 |
| Age | 40 | Continuous | 0.316 |
| Contralateral SLR movement | 40 | Continuous | 0.517 |
| Short form McGill Pain Questionnaire - total score | 40 | Continuous | 0.59 |
| Oswestry score | 40 | Continuous | 0.619 |
| Extension ROM | 40 | Continuous | 0.723 |
| Ipsilateral SLR movement | 40 | Continuous | 0.874 |
| Pain location | 40 | NOMINAL (multi) | 0.122 |
| MRI - Nerve root contact | 39 | NOMINAL (multi) | 0.138 |
| MRI - bulge type | 39 | NOMINAL (multi) | 0.175 |
| MRI - canal compromise by disc herniation | 39 | NOMINAL (multi) | 0.366 |
| Mechanism of injury | 40 | NOMINAL (multi) | 0.421 |
| Time of day of injury / first onset | 40 | NOMINAL (multi) | 0.574 |
| Time of day when pain worst | 40 | NOMINAL (multi) | 0.632 |
| MRI - Annular tear | 39 | NOMINAL (multi) | 0.893 |
